# RETRACTION: Exploration of Wound‐Related Complications Post‐Kidney Transplantation

**DOI:** 10.1111/iwj.70485

**Published:** 2025-04-02

## Abstract

**RETRACTION:** SunH. , LiuK. , PengZ. , and LiuS. , “Exploration of Wound‐Related Complications Post‐Kidney Transplantation,” International Wound Journal 21, no. 4 (2024): e14578, 10.1111/iwj.14578.38113325 PMC10961868

The above article, published online on 19 December 2023, in Wiley Online Library (http://onlinelibrary.wiley.com/), has been retracted by agreement between the journal Editor in Chief, Professor Keith Harding; and John Wiley & Sons Ltd. Following an investigation by the publisher, all parties have concluded that this article was accepted solely on the basis of a compromised peer review process. In addition, the authors provided incomplete ethical approval information for the study. The editors have therefore decided to retract the article. The authors did not respond to our notice regarding the retraction.

**RETRACTION:** H. Sun , K. Liu , Z. Peng , and S. Liu , “Exploration of Wound‐Related Complications Post‐Kidney Transplantation,” International Wound Journal 21, no. 4 (2024): e14578, 10.1111/iwj.14578.38113325 PMC10961868

The above article, published online on 19 December 2023, in Wiley Online Library (http://onlinelibrary.wiley.com/), has been retracted by agreement between the journal Editor in Chief, Professor Keith Harding; and John Wiley & Sons Ltd. Following an investigation by the publisher, all parties have concluded that this article was accepted solely on the basis of a compromised peer review process. In addition, the authors provided incomplete ethical approval information for the study. The editors have therefore decided to retract the article. The authors did not respond to our notice regarding the retraction.

